# Paricalcitol Reduces Peritoneal Fibrosis in Mice through the Activation of Regulatory T Cells and Reduction in IL-17 Production

**DOI:** 10.1371/journal.pone.0108477

**Published:** 2014-10-03

**Authors:** Guadalupe T. González-Mateo, Vanessa Fernández-Míllara, Teresa Bellón, Georgios Liappas, Marta Ruiz-Ortega, Manuel López-Cabrera, Rafael Selgas, Luiz S. Aroeira

**Affiliations:** 1 Research Department, Instituto de Investigación Sanitaria del Hospital Universitario La Paz (IdiPAZ), Hospital La Paz, Madrid, Spain; 2 Molecular Biology Center Severo Ochoa, CSIC-UAM, Madrid, Spain; 3 Cellular Biology in Renal Diseases Laboratory, IIS-Fundación Jiménez Díaz/Autonomous University of Madrid, Madrid, Spain; Red de Investigación Renal REDinREN, Madrid, Spain; 4 Nephrology Department, Hospital Universitario La Paz, Madrid, Spain; Red de Investigación Renal REDinREN, Madrid, Spain; Instituto Reina Sofía de Investigación en Nefrología (IRSIN), Madrid, Spain; 5 Immunology Department, Centro de Investigaciones Biomédicas (CINBIO), Instituto de Investigación Biomédica de Vigo (IBIV), University of Vigo, Vigo, Spain; Nihon University School of Medicine, Japan

## Abstract

Fibrosis is a significant health problem associated with a chronic inflammatory reaction. The precise mechanisms involved in the fibrotic process are still poorly understood. However, given that inflammation is a major causative factor, immunomodulation is a possible therapeutic approach to reduce fibrosis. The vitamin D receptor (VDR) that is present in all hematopoietic cells has been associated with immunomodulation. We investigated whether the intraperitoneal administration of paricalcitol, a specific activator of the VDR, modulates peritoneal dialysis fluid (PDF)-induced peritoneal fibrosis. We characterized the inflammatory process in the peritoneal cavity of mice treated or not treated with paricalcitol and analyzed the ensuing fibrosis. The treatment reduced peritoneal IL-17 levels, which strongly correlated with a significantly lower peritoneal fibrotic response. *In vitro* studies demonstrate that both CD4^+^ and CD8^+^ regulatory T cells appear to impact the regulation of IL-17. Paricalcitol treatment resulted in a significantly increased frequency of CD8^+^ T cells showing a regulatory phenotype. The frequency of CD4^+^ Tregs tends to be increased, but it did not achieve statistical significance. However, paricalcitol treatment increased the number of CD4^+^ and CD8^+^ Treg cells *in vivo*. In conclusion, the activation of immunological regulatory mechanisms by VDR signaling could prevent or reduce fibrosis, as shown in peritoneal fibrosis induced by PDF exposure in mice.

## Introduction

Tissue fibrosis is the consequence of abnormal healing processes that are typically associated with chronic inflammation, and is an important cause of morbidity and mortality. The molecular and cellular mechanisms that contribute to the development of fibroproliferative diseases are still poorly understood, hindering the development of efficient therapies. Understanding the specific mechanisms involved could contribute to the development of therapeutic procedures.

Peritoneal dialysis (PD) is a renal replacement therapy for patients with end-stage renal disease (ESRD). This therapy uses the peritoneum as a semi-permeable membrane, exposing it to a bio-incompatible, glucose-containing hyperosmotic solution known as peritoneal dialysis fluid (PDF). When chronically exposed to PDF, patients develop peritoneal fibrosis and ultrafiltration failure, which compromises treatment efficacy and patient outcomes [1]. The mesothelial cell monolayer is the first line of contact between the PDF and the body, and could trigger a peritoneal aseptic inflammatory process. The exposure of mesothelial cells to glucose degradation products (GDPs), Amadori adducts and advanced glycation end products (AGEs) stimulated NF-κB-mediated transcription and the secretion of cytokines and chemokines *in vitro* [2], [3], [4]. PD results in increased serum concentrations of IL-6, TNF-α, VEGF and C-reactive proteins in patients, which suggests that PD leads to increased systemic inflammation [5], [6]. Using a mouse model of chronic peritoneal exposure to PDF, we had already demonstrated that PDF induces peritoneal membrane inflammation and fibrosis [7]. The use of an anti-inflammatory drug (a Cox-2 inhibitor) reduced peritoneal inflammation and fibrosis [8]. This result confirms the role of inflammation in peritoneal fibrosis. However, the cardiovascular effects of this drug family preclude its use in patients undergoing PD.

In addition to its classic metabolic action, vitamin D has an immunomodulatory function. The vitamin D receptor (VDR) is a nuclear hormone receptor expressed in B, T, macrophages and dendritic cells, and it regulates their proliferation and function [9]. Vitamin D can prevent or ameliorate inflammatory disease in animal autoimmune disease models [10] and in patients [11]. The mechanism by which vitamin D regulates inflammatory response appears to be dependent on the activation of regulatory CD4^+^ T cells [12]. These regulatory CD4^+^ T cells control IL-17 and IFN-γ secretion [13], [14], [15]. Because inflammation plays an important role in peritoneal fibrosis, vitamin D receptor (VDR) signaling might have a beneficial impact on PDF-induced peritoneal fibrosis. Paricalcitol is a selective VDR activator used to treat secondary hyperparathyroidism, a disorder often seen in patients with chronic renal failure. As a VDR activator, paricalcitol has shown anti-inflammatory activity [16]. Therefore, given its various therapeutic targets, paricalcitol could represent a very interesting treatment for patients undergoing PD. To address the impact of VDR signaling on peritoneal fibrosis, paricalcitol was added to a commercial PDF, and its effect on PDF-induced peritoneal inflammation and fibrosis was addressed in a mouse model of peritoneal dialysis. The results demonstrate that paricalcitol prevents peritoneal membrane deterioration, reducing fibrosis and ultrafiltration failure. The mechanism of protection appears to be dependent on the increased frequency of regulatory T cells and the reduction in IL-17 production in the peritoneal cavity.

## Materials and Methods

### Animals and surgery

The experimental procedure was approved by the ethics committee for animal welfare of La Paz University Hospital (CEBA 06-2009). Female C57BL/6 mice, from 12–14 weeks of age, were purchased from Charles River (Barcelona, Spain) and maintained in conventional conditions in our animal facilities. Catheter implantation was performed as previously described [8]. The state of the animals' health was checked daily by a veterinarian, and mice presenting any sign of illness were excluded from the experiment. Only 3 mice were excluded from the experiment. The cause of exclusion was an infection at the site of the subcutaneous pump.

We used 3 groups of mice in these experiments. These groups were repeated in duplicate to analyze both the ultrafiltration capacity of the peritoneum and the production of cytokines and chemokines. These two measurements were not performed together because they required technical differences during euthanasia. Thereby, the experiments were performed in duplicate, and were repeated at least twice. The control group had a catheter and received no PDF (n = 6). This group was used to demonstrate that a catheter alone does not affect the peritoneal membrane. The PDF group received an instillation of 2 ml of staysafe (4.25% glucose) PDF (Fresenius Medical Care, Germany) daily through the catheter (n = 10). The paricalcitol group received 2 ml of staysafe (4.25% glucose) supplemented with paricalcitol daily (n = 11). The mice were treated for 30 days. At the end of the experiment, the mice were visually checked for the presence of peritoneal infection (signs of pus at the peritoneal cavity), and peritoneal washing fluid from the 3 mouse groups was seeded in agar plates for bacterial culture; no bacterial infection was detected. To study the mechanisms involved in paricalcitol peritoneal protection, we performed only the comparison between the PDF and the paricalcitol groups.

The experimental procedure was performed according to NIH guidelines for the care and use of laboratory animals and was approved by the institutional ethics committee.

### Paricalcitol treatment

Paricalcitol dissolved in PDF was instilled daily into the mouse peritoneal cavity for 30 days, at a dosage of 0.3 µg/kg/week (0.86 ng/mouse/day). The dose used in mice is low considering the translation to humans based on body surface area [17], which would mean that human patients should receive about 0.024 µg/kg/week, whereas they normally receive 0.117 µg/kg/week.

### Histological analyses and immunofluorescence

Parietal peritoneal biopsies were collected from the opposite side of the catheter installation. Part of the biopsies were fixed in Bouin's solution, embedded in paraffin, cut into 5 µm sections and stained with Masson's trichrome. The peritoneal membrane thickness was determined using a microscope (Leica CTR6000, with a Leica Microsystems LAS-AF6000). Microphotographs were obtained using an Olympus BX41 clinical microscope and an Olympus DP20 digital camera using cell acquisition software.

The peritoneal thickness of each mouse was calculated by the median of measurement taken every 50 µm from one extreme to the other of the biopsy. The result was used to calculate the group thickness.

For the immunofluorescence analysis, other biopsies were frozen in optimal cutting temperature (OCT) compound and cut into 5 µm sections. To identify the mesothelial cells, a mouse anti-cytokeratin 8/18 (clone 5D3 Novocastra, Newcastle, UK) antibody was used, which was stained with anti-IgG1 specific Zenon Fab fragments (Invitrogen, Eugene, OR, USA) according to the manufacturer's instructions. The blood vessels were stained with anti-CD31 and Zenon Fab. The CD45^+^ cells were identified using a rat anti-mouse CD45 (BD Bioscience) and were stained with goat anti-rat antibodies. To stain CD4^+^ and CD8^+^ cells, the same antibodies used in flow cytometry were employed. The nuclei were stained with DAPI. The micrography was performed with a fluorescence microscope (Leica CTR6000, Leica Microsystems Heidelberg, Germany, with LAS-AF6000 software) or with a confocal microscope (Leica TCS SPE with LAS-AF software, version 2.0.1 build 2043).

### Net ultrafiltration

On the final day of the experiment, the mice were euthanized 40 minutes after receiving 2 ml of PDF, and the total peritoneal volume was drained and measured.

### Chemokine and cytokine detection

To determine the peritoneal production of cytokines and chemokines, the peritoneal cavity was washed in this case with 2 ml of saline immediately after the mice were euthanized. The molecule concentration is thereby not affected by the differences in ultrafiltration. The solution was centrifuged, and the supernatant was separated into small aliquots and stored at -80°C. The peritoneal chemokines and cytokines were determined using the FlowCytomix technique (Bender MedSystems GmbH).

### Flow cytometry analyses

The peritoneal cells were analyzed by flow cytometry using the following antibodies purchased from BD Biosciences (San Diego, CA, USA): anti-mouse CD4 PE, anti-mouse CD3e biotin, anti-mouse CD8a Alexa Fluor 488-labeled and anti-Foxp3 Alexa 647. FoxP3 stainning was performed according to the manufacturer's recommendations in the kit “Mouse FoxP3 Buffer set” (BD Pharmingen).

The biotin-conjugated anti-CTLA4 and PE-Cy7-conjugated streptavidin were acquired from eBioscience (San Diego, USA). The flow cytometry analysis was conducted on a FACSCalibur flow cytometer (BD Biosciences) using Cell Quest Pro software.

### Determination of IL-17 producing cells

Peritoneal cells from the PDF-treated mice were enriched in T cells by negative selection using magnetic beads coated with monoclonal antibodies against unrelated populations. The elimination of bead linked cells was performed in an autoMACS cell separator (Miltenyi Biotec) according to the manufacturer's instructions. These cells enriched in T lymphocytes were used as the “effector T cell” population. Meanwhile, cells from the PDF or paricalcitol-treated groups were divided and enriched in CD4^+^ or CD8^+^ T cells through negative selection of irrelevant cells to analyze their regulatory capacity. These CD4^+^ and CD8^+^ enriched T cell populations were stained with PKH67 (PKH67 green fluorescent linker; Sigma Aldrich) and cocultured with the enriched T cell population from the PDF-treated mice, at a ratio 1∶1(75×10^3^ cells of each population). A negative control without the CD4^+^ or CD8^+^ enriched T cell populations was also analyzed.

The cells were cultured for 2 days in a 96-well plate pre-coated for 24 h at 4°C with anti-CD3 antibody (purified hamster anti-mouse CD3e; BD Biosciences) and activated with anti-CD28 (2 µg/ml) antibody (purified hamster anti-mouse CD28; BD Biosciences). On the final day, the cells were exposed to brefeldin A (2.5 µg/mL) for 12 h, and the expression of intracellular IL-17 was then analyzed by flow cytometry. Finally, the frequency of the IL-17^+^ CD4^+^ T cells from the PKH67^negative^ population was determined.

### Statistical analysis

The data were analyzed using GraphPad Prism (version 4) (La Jolla, CA, USA) software for Macintosh, and the results were presented in box plot graphics. The bottom and top of the box represent the first and third quartiles, and the median is represented by the band inside the box. The ends of the whiskers represent minimum and maximum values. Comparisons between the control and the paricalcitol groups were performed using the nonparametric Mann-Whitney U test. A value of *P*<.05 was considered statistically significant. All the experiments were repeated at least twice. The correlation analysis was performed using Spearman's nonparametric test.

## Results

### Paricalcitol treatment ameliorated peritoneal inflammation and fibrosis

**Figure 1:**
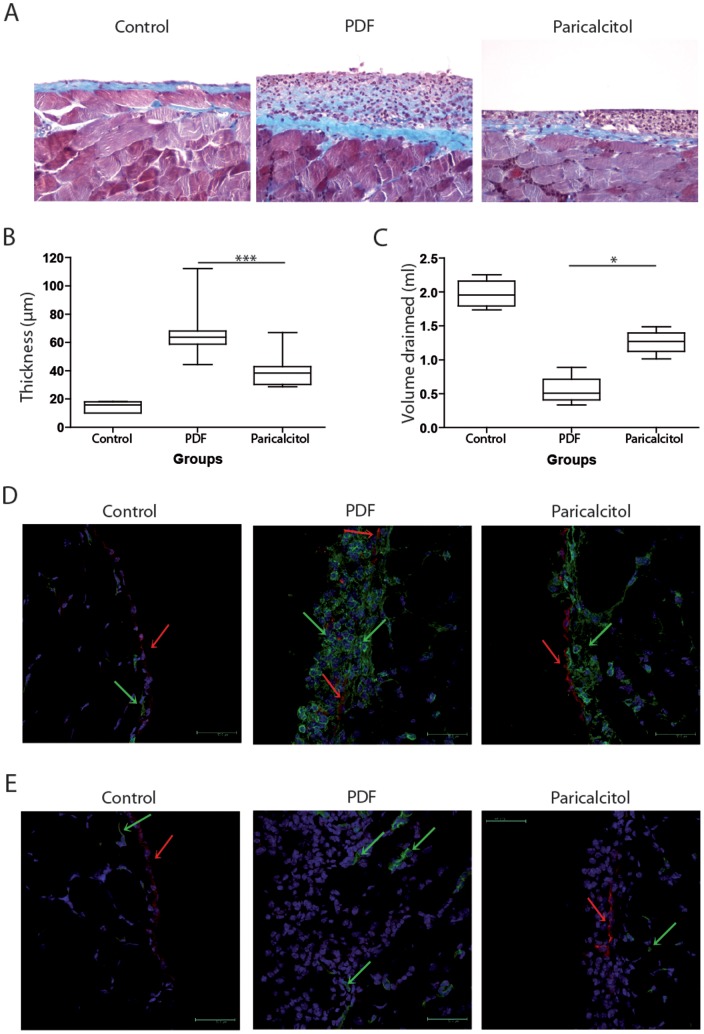
Paricalcitol reduced peritoneal membrane fibrosis, inflammation and ultrafiltration failure in mice exposed to PDF. A) Paraffin sections of the peritoneal membrane from the 3 groups were stained with Masson's trichrome. B) Thickening of the peritoneal membrane was determined by morphometric analysis. C) Peritoneal permeability was determined by net ultrafiltration. D) The presence of inflammatory and mesothelial cells was determined by the expression of CD45 (green) and cytokeratin (red), respectively, in frozen sections of peritoneal membrane representative of each group. A green arrow indicates hematopoietic cells. A red arrow indicates mesothelial cells. E) The angiogenesis was determined by the expression of CD31 (green). Cytokeratin-positive cells are stained in red. The color balance was equally adjusted in immunofluorescence using Photoshop V10 for Mac (Abobe Systems Incorporated, US). n≥5 in each group. Statistical significance was determined using the Mann-Whitney test. *P<.05; ***P<.001.

PDF treatment was shown to induce peritoneal fibrosis in mice. To evaluate the effect of VDR signaling on peritoneal fibrosis, intraperitoneal paricalcitol was administered daily with PDF in an independent group of mice, and the thickness of peritoneal membrane was evaluated as a measure of peritoneal fibrosis. Masson's trichrome staining of the peritoneal biopsies revealed that the paricalcitol group had less matrix deposition and cell infiltration in the peritoneal membrane than the PDF group (Figure 1A). This was confirmed by a morphometric analysis, which showed thicker peritoneal membranes in the PDF-treated group as compared with the paricalcitol-treated group (Figure 1B). Increased thickness was the consequence of cellular and matrix deposition over the peritoneal membrane.

A functional analysis of the peritoneal membranes revealed that the paricalcitol group had better ultrafiltration than the PDF group (Figure 1C, *P*<.05). This result might be the consequence of paricalcitol's effect on inflammation and angiogenesis. In the peritoneal membranes of the PDF group, CD45^+^ cells were found on both sides of the mesothelial cell layer, which was not well preserved. The mesothelial cells of the paricalcitol-treated mice were better preserved than in the PDF group, and CD45^+^ cells were present in lower numbers and located primarily in the sub-mesothelial space (Figure 1D).

One of the key events in tissue regeneration is angiogenesis at the fibrotic area generating granulose tissue. In PD-treated patients, this is related to ultrafiltration failure. This effect was also found in PDF-treated mice, which presented blood vessels embedded in the inflammatory zone. In addition, these mice had increased numbers of blood vessels in the muscular package at the base of the peritoneal membrane as compared with the control mice. The paricalcitol group had fewer blood vessels at the base and no blood vessels embedded in the inflammatory zone (Figure 1E).

### Paricalcitol treatment increased T cell populations in the peritoneal cavity

**Figure 2:**
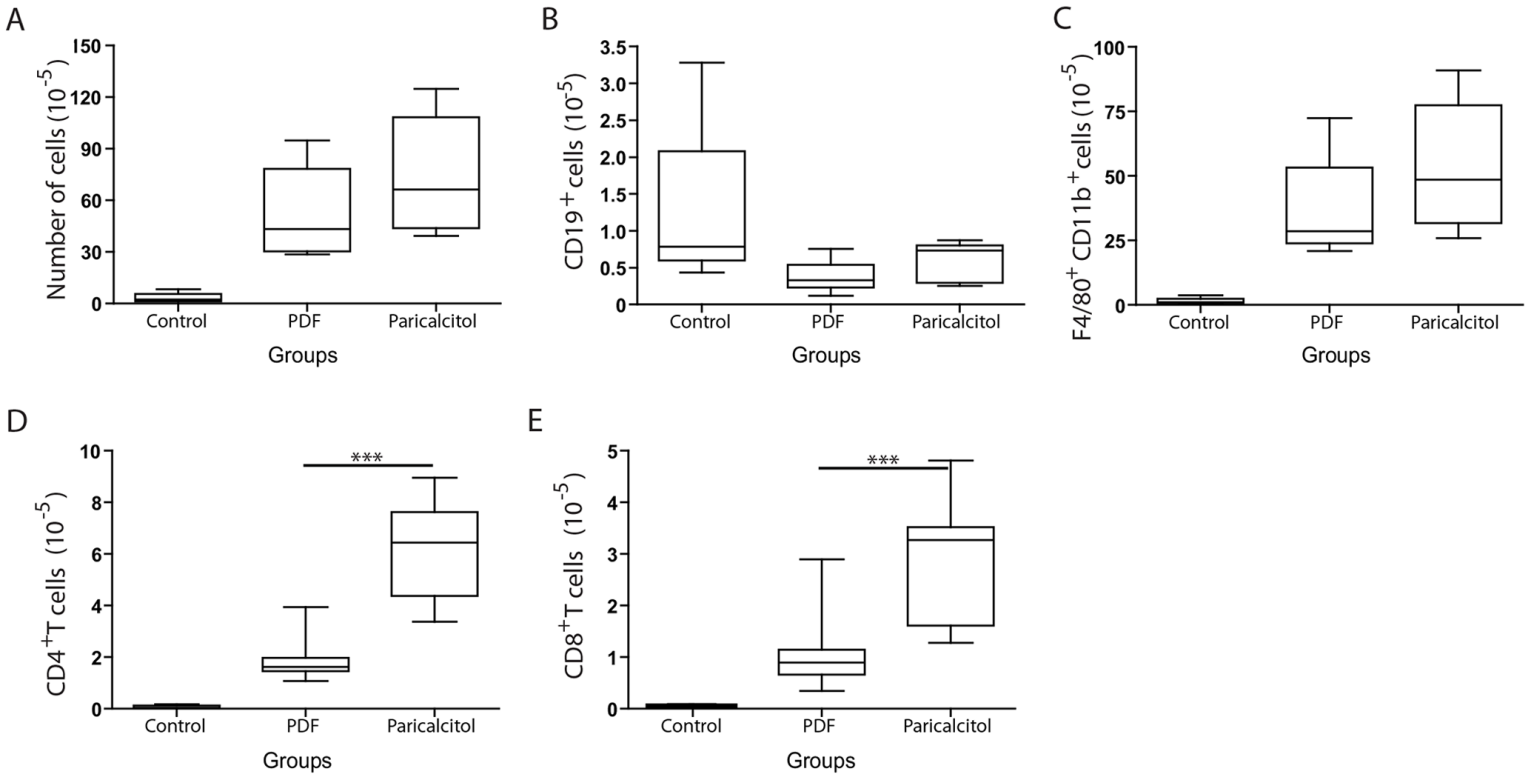
Paricalcitol changed the peritoneal T cell population in mice instilled with PDF. A) The paricalcitol group tends to increase the number of nucleated cells at the peritoneal cavity compared with the PDF group. B) The number of B cells was similar in the PDF and paricalcitol groups. C) There is no significant difference between the number of macrophages in the PDF and paricalcitol groups. D) Paricalcitol increased the numbers of CD4^+^ T cells compared with the PDF group. E) The treatment with paricalcitol increased CD8^+^ T cells in the peritoneal cavity. Statistical significance was determined using the Mann-Whitney test. ***P<.001; n≥5 in each group.

We next analyzed the phenotype of the leukocytes present within the peritoneal cavity after treatment. The paricalcitol-treated mice tended to have a higher number of nucleated cells (Figure 2A); however, the number of B cells in the control group was higher than in the PDF group and similar to that observed in the paricalcitol group (Figure 2B). The PDF- and paricalcitol-treated mice presented a higher number of total macrophages (Figure 2C), and the number of B cells and macrophages were similar between both groups (Figure 2B and 2C, respectively). The PDF and paricalcitol groups had a higher number of T cells as compared with the control group. However, the paricalcitol group had increased numbers of CD4^+^ (Figure 2D) and CD8^+^ (Figure 2E) T cells as compared with the PDF group.

**Figure 3:**
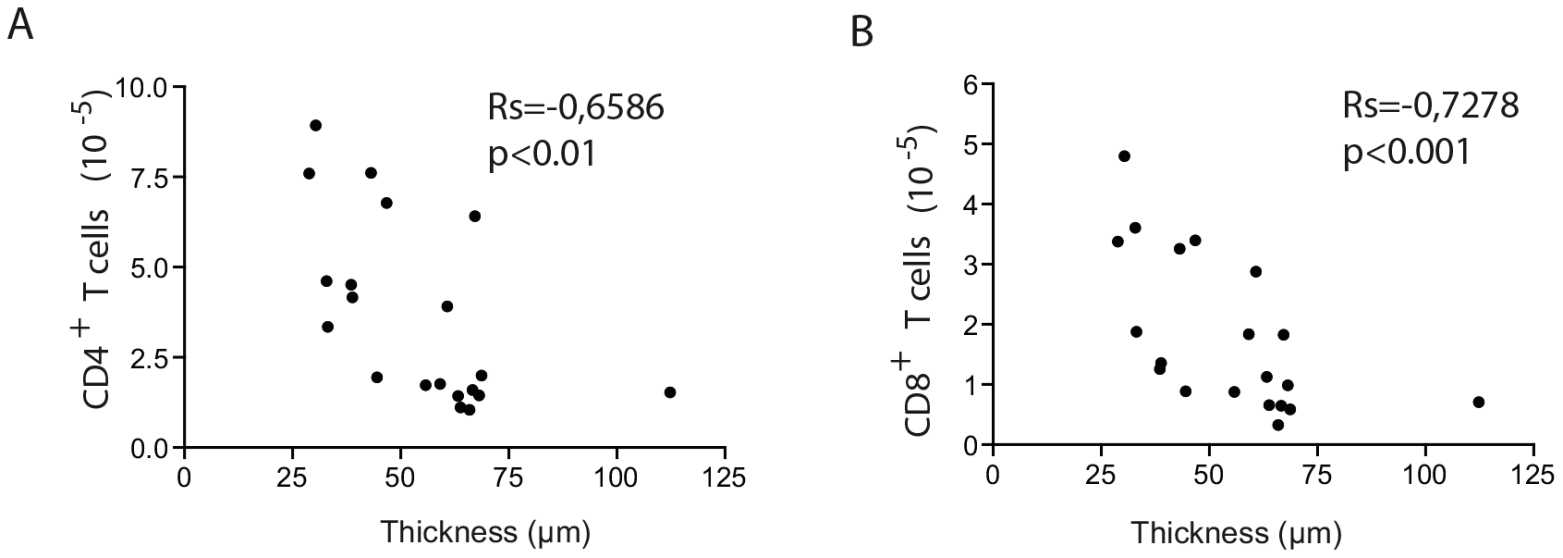
Paricalcitol's enhancement of T cell numbers is inversely correlated with peritoneal thickness. The increased levels of CD4^+^ (A) and CD8^+^ (B) T cells are strongly correlated with lower peritoneal fibrosis. n≥5 in each group. Statistical significance was determined using the Mann-Whitney test. A correlation analysis was performed using Spearman's nonparametric test.

The numbers of CD4^+^ and CD8^+^ T cells in the mice exposed to PDF and treated or not treated with paricalcitol were inversely correlated with peritoneal membrane thickness (Figure 3). This result strongly suggests that the effect of paricalcitol treatment on T cells might play a role in the prevention of peritoneal fibrosis.

### Paricalcitol increased the number of T cells expressing a regulatory phenotype in the peritoneal cavity

**Figure 4:**
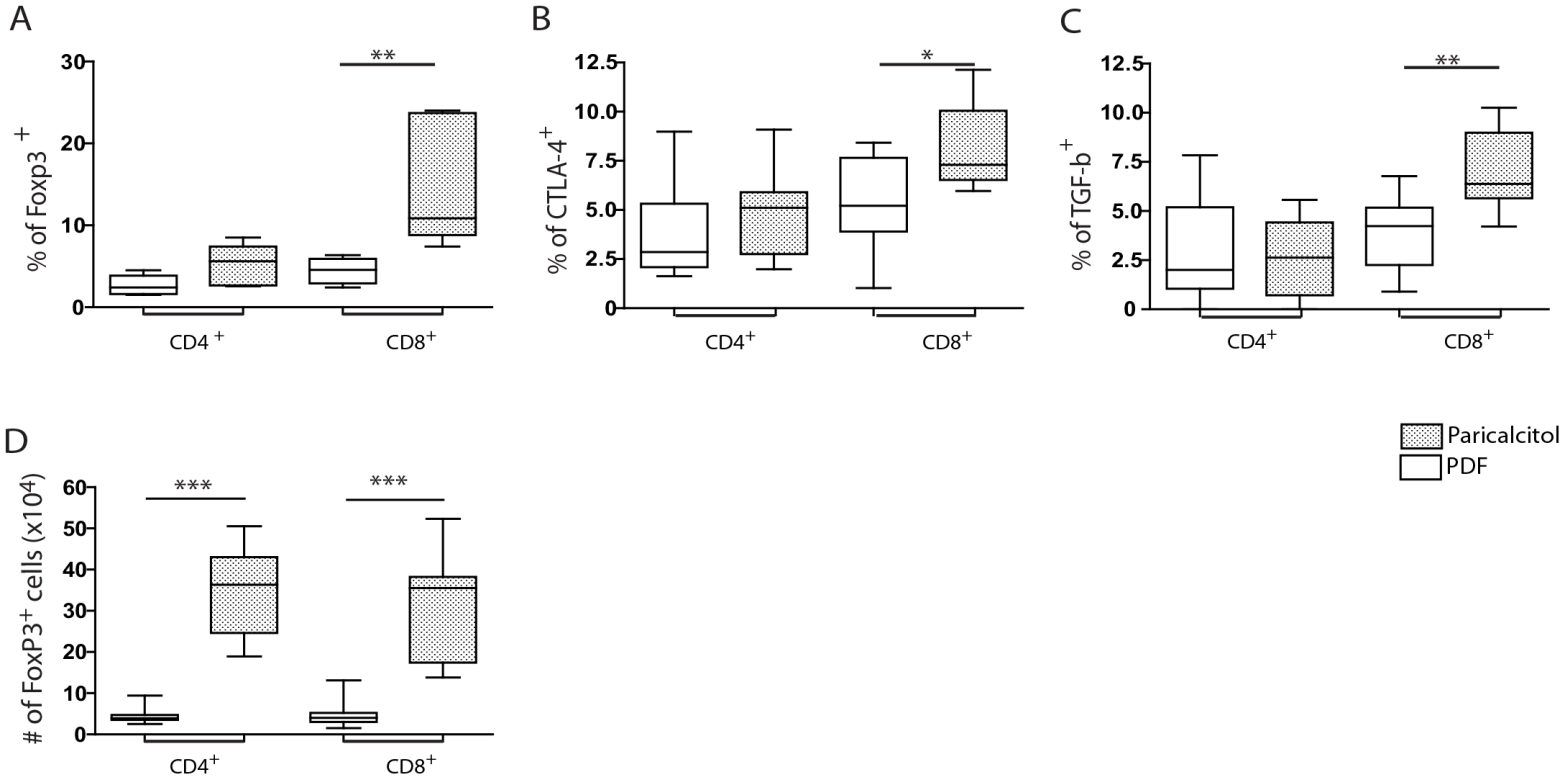
Paricalcitol induced the recruitment of regulatory CD8+ T cells into the peritoneal cavity. Paricalcitol (dashed box) tended to increase the frequency of CD4^+^ T cells expressing Foxp-3 (A), but not CTLA-4 (B) or membrane TGF-β (C). Paricalcitol increased the frequency of CD8^+^ T cells expressing Foxp-3 (A), CTLA-4 (B) and membrane TGF-β (C) compared with the PDF-group (white box). n≥9 in each group. Paricalcitol treated group had higher number of CD4^+^ CD8^+^ T cells expressing Foxp-3 than PDF group (D). Statistical significance was determined using the Mann-Whitney test. *P<.05; **P<.01; ***P<.001.

Vitamin D has previously been associated with the activation and proliferation of CD4^+^ T regulatory cells [18]. Regulatory T cells are characterized by the expression of Forkhead P3 (Foxp3) transcription factor and the membrane antigens cytotoxic T-lymphocyte antigen-4 (CTLA-4) and TGF-β. These cells are responsible for the regulation of the immune response. The paricalcitol-treated mice tended to have higher frequency of Foxp-3^+^CD4^+^ T cells than that observed in the PDF group. However, the frequency of CD8^+^ T cells expressing Foxp-3 was significantly higher in the paricalcitol-treated mice than in the PDF-treated mice (Figure 4A). Both the paricalcitol and the PDF groups expressed similar frequencies of CD4^+^ T cells expressing CTLA-4^+^ and membrane TGF-β (Figure 4B and 4C, respectively), whereas both markers were expressed at higher levels in the CD8^+^ T cells of the paricalcitol-treated mice than in those of the PDF group. These results clearly show that paricalcitol preferentially improves the frequency of CD8^+^ Treg in the peritoneal cavities of mice exposed to PDF. Nonetheless, the absolute number of CD4^+^ and CD8^+^ T regulatory cells (FoxP3^+^) were higher in animals treated with paricalcitol (Figure 4D).

### Paricalcitol treatment did not interfere with chemokine production

**Figure 5:**
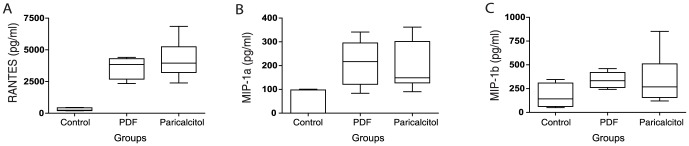
Chemokine concentrations in the peritoneal cavity were not affected by paricalcitol treatment. Concentrations of RANTES (A), MIP-1α (B) and MIP-1β (C) were determined in the peritoneal washing fluid. We observed no difference between the paricalcitol and the PDF group. n≥5 in each group.

The levels of RANTES, MIP-1α and MIP-1β (Figs. 5A–5C, respectively) were similar in both the paricalcitol and the PDF groups. The results suggest that paricalcitol treatment did not interfere with chemokine production.

### Paricalcitol treatment reduced IL-17 concentrations in the peritoneal cavity

**Figure 6:**
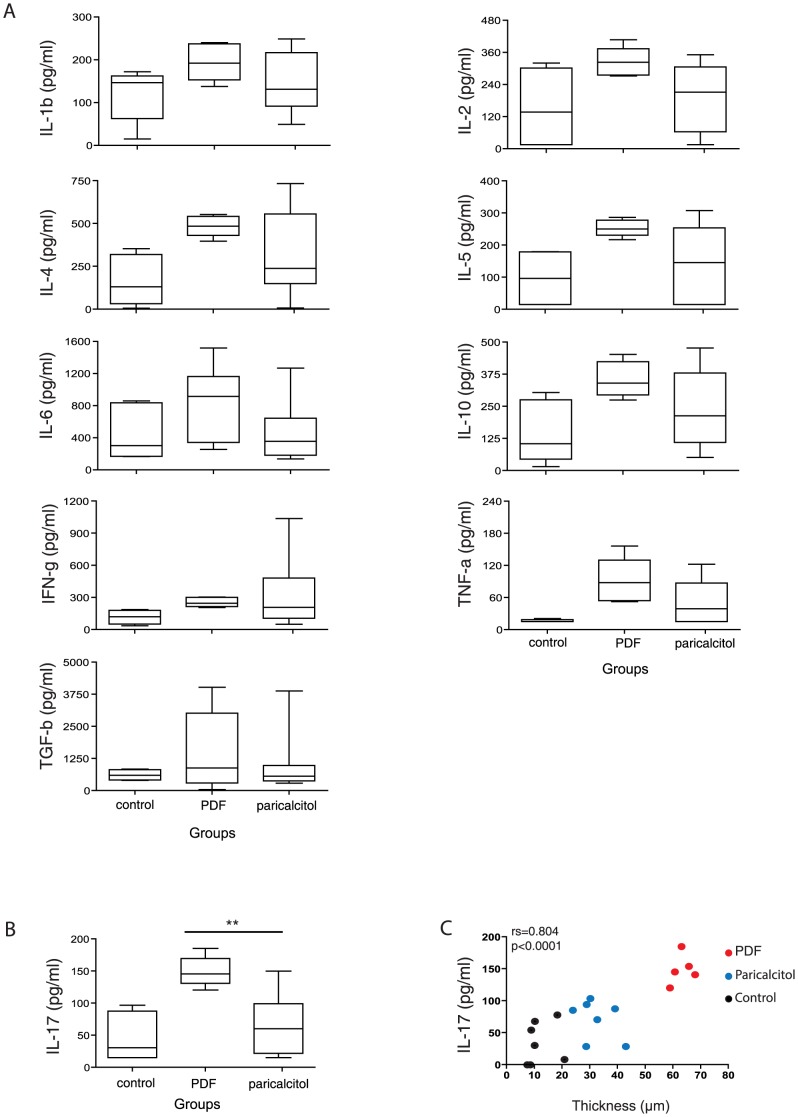
Paricalcitol induced the regulation of IL-17 production and affected peritoneal fibrosis outcomes. A) Peritoneal concentrations of IL-1β, IL-2, IL-4, IL-5, IL-6, IL-10, IFN-γ and TNF-α were not affected by paricalcitol treatment. B) Paricalcitol induced the suppression of IL-17 production in the peritoneal membrane. C) The IL-17 concentrations strongly correlate with peritoneal fibrosis. n≥5 in each group. Statistical significance was determined using the Mann-Whitney test. **P<.01. A correlation analysis was performed using Spearman's nonparametric test.

Due to the fact that cytokines play a role in inflammation and that Tregs regulate their production, the concentration of several cytokines in the peritoneal cavity was determined. The results showed that paricalcitol did not significantly reduce the concentrations of IL-1β, IL-2, IL-4, IL-5, IL-6, IL-10, TNF-α, IFN-γ and TGF-β compared with the PDF group (Figure 6A). However, the PDF group presented significantly higher levels of IL-17 than the paricalcitol group (*P*<.01) (Figure 6B). Furthermore, IL-17 levels were strongly correlated with peritoneal thickness (Figure 6C), suggesting that IL-17 plays an important role in the development of peritoneal fibrosis and that the prevention of fibrosis by paricalcitol might be directly linked to the downregulation of IL-17 cytokine levels.

### Peritoneal CD4^+^ and CD8^+^ T cells from the paricalcitol-treated mice reduced the production of IL-17 production by in vitro-stimulated T cells

**Figure 7:**
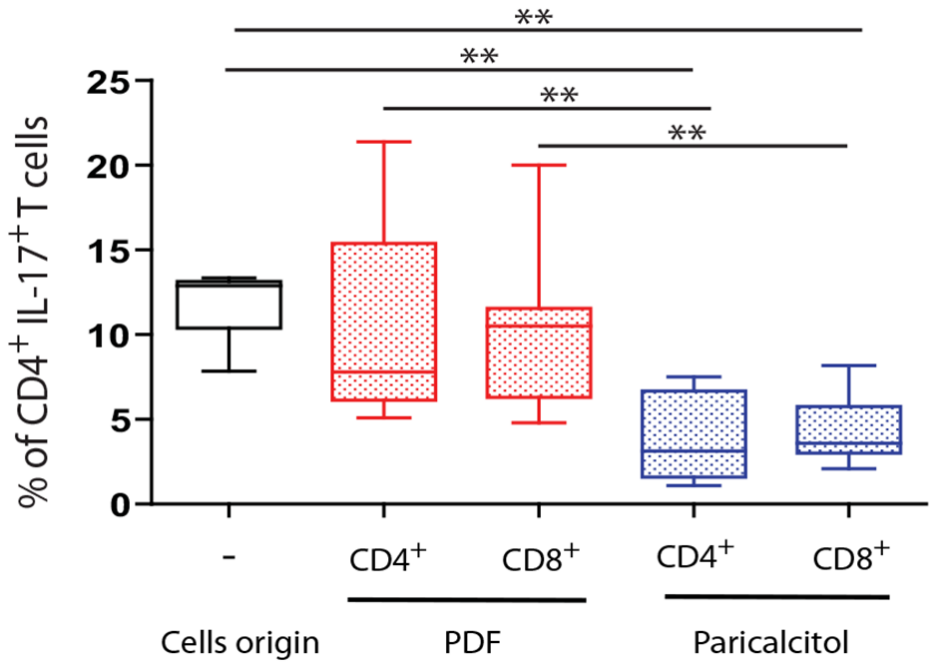
CD4+ and CD8+ T cells from paricalcitol-treated mice regulate IL-17 production. CD4^+^- and CD8^+^-enriched T cell populations from mice treated with PDF and paricalcitol were stained with PKH67 and co-cultured with CD3^+^ T cells from PDF-treated mice. A control group of CD3^+^ T enriched cells from PDF-treated mice was also included. The cells were stimulated with CD3 and CD28, and after 2 days, brefeldin A was added for the last 12 h of culture. The cells were stained with anti-CD4 and anti-IL-17 antibodies and analyzed by flow cytometry. The PKH67^negative^ CD4^+^ T cells were selected, and the frequency of IL17^+^ cells is shown in the figure. Statistical significance was determined using the Mann-Whitney test. **P<.01.

Given that regulatory T cells could affect the production of IL-17, we investigated if peritoneal CD4^+^ and CD8^+^ T cells from mice treated or not with paricalcitol can regulate the production of IL-17 by *in vitro*-stimulated CD4^+^ T cells. For this purpose, a cell population enriched in CD4^+^ or CD8^+^ T cells from PDF- or paricalcitol-treated mice was stained with PKH67 and cocultured in the presence of peritoneal CD3^+^ T cells from mice treated with PDF. The cultures were stimulated with anti-CD3 and anti-CD28 antibodies, and the frequency of CD4^+^IL-17^+^ T cells in the PKH67^negative^ population was determined. The results in Figure 7 show that both peritoneal CD4^+^ and CD8^+^ T cells-enriched populations from paricalcitol-treated mice reduced the frequency of IL-17^+^CD4^+^ T cells obtained from the peritoneum of mice treated only with PDF. This frequency was significantly lower than that observed when analyzing CD4^+^ or CD8^+^ T cells-enriched populations from PDF-treated mice.

## Discussion

Fibrosis is the final consequence of a chronic inflammatory reaction [19], [20], [21]. Therefore, regulation of the inflammatory reaction could have beneficial effects on fibrotic disorders. VDR signaling has previously been shown to regulate inflammation [18] and has shown fibroprotective activity in various organs [22], [23], [24]. Various mechanisms have been proposed to explain the role of VDR signaling in fibrosis regulation, but it is still poorly understood. The activation of this pathway has been shown to inhibit myofibroblast activation [25], to induce differentiation of mesenchymal precursors into a non-fibrogenic phenotype [26] and to reduce inflammation through NF-κB sequestration [16]. These mechanisms are not mutually exclusive and might work together.

Inflammation is a key event in wound healing and fibrosis [27]. It is known that macrophages play an important role in the fibrotic process through the secretion of growth factors such as TGF-β and FGF. Here we addressed the role of paricalcitol in the development of fibrosis using a mouse model of peritoneal dialysis. Given that inflammation plays an important role in fibrosis, we centered our attention on the inflammatory mechanisms.

As previously reported by our group [8], peritoneal exposure to PDF induced increased numbers of inflammatory cells at the peritoneal cavity, composed primarily of macrophages. However, the number of CD4^+^ and CD8^+^ T cells in the peritoneal cavity of mice exposed to PDF increased as well. T cells are responsible for the orchestration of inflammatory responses through the secretion of cytokines, which also play a role in fibrosis by the activation of fibroblasts [28], [29] and fibrocytes [30]. Th2 responses are associated with fibrosis development, whereas Th1 responses are associated with anti-fibrotic actions [20], [31]. However, Th1 type responses have also been implicated in peritoneal fibrotic adhesion [32] and autoimmune-induced cardiac fibrosis [33], whereas Th17 responses have been related to fibrosis in the kidneys [34], lung [35] and skin [36]. We have recently demonstrated that IL-17 plays an important role in the generation of PDF-induced peritoneal fibrosis in patients and mice exposed to PDF [37]. CD4^+^ T cells were the primary source of IL-17 in the peritoneum of the mice treated with PDF. We have also demonstrated that the direct administration of recombinant IL17 in the peritoneum induced peritoneal fibrosis in mice [37]. These results reveal that, depending on the model studied, different T cell phenotypes appear to be potentially fibrogenic.

Several studies have assessed the cytokine pattern produced in the peritoneal cavity of patients undergoing PD, with conflicting results [38], [39], [40]. The discrepancy in these results could be explained by the *in vitro* experimental approaches used. The PD mouse model, contrary to expectations, revealed an upregulation of all Th responses. This could be explained by the fact that approaches using *in vitro* cell stimulation might induce biases in cytokine production depending on the methodology used, whereas for the PD mouse model, cytokines are produced *in vivo* in response to PDF instillation.

Previous studies have demonstrated that VDR activation induces an increased frequency of CD4^+^ regulatory T cells [18]. In this study, paricalcitol treatment tended to increase the frequency of CD4^+^ T cells expressing Foxp3 (*P* = .054), whereas the impact on the expression of other Treg markers was less important. The treatment with paricalcitol, however, increased the frequency of peritoneal CD8^+^ T cells expressing Foxp-3, CTLA-4 and membrane TGF-β. These results suggest that, at least in this model, VDR signaling preferentially affects CD8^+^ T cells. This selective effect on CD8^+^ T cells could be explained by the fact that mature CD8^+^ T cells express higher concentrations of VDR than CD4^+^ T cells [41]. To our knowledge, this was the first time that a preferential effect on the activation/development of regulatory CD8^+^ T cells was demonstrated *in vivo*.

As shown in Figure 3, the number of CD4^+^ and CD8^+^ T cells is higher in paricalcitol-treated mice than in PDF mice. As a result of the increased number of CD4^+^ T cells in paricalcitol and the tendency towards and increased frequency of CD4^+^ T cells expressing Foxp3, the number of peritoneal CD4^+^ Treg cells was higher in paricalcitol than in PDF treated mice (P<.001). Similarly, the number of peritoneal CD8^+^ T cells expressing Foxp3 was higher in paricalcitol-treated than in PDF mice. This outcome strongly suggests that the increased number of regulatory T cells is responsible for inflammation regulation in the peritoneal cavity.

The majority of studies concerning T cell-mediated regulation of immune response have been focused on CD4^+^ T cells. The existence of CD8^+^ T cells with regulatory activity (called “suppressor” cells) was demonstrated during the 1970s and 1980s, however, the study of these cells was hindered by technical limitations. The interest in CD8^+^ regulatory T cells has recently been revived, and increasing evidence points to their participation in the control of various inflammatory processes [42] through the regulation of IL-17 and IFN-γ. The results presented here show that paricalcitol treatment selectively reduced IL-17 peritoneal concentration. The regulation of IL-17 production was likely mediated by both CD4^+^ and CD8^+^ T cells from paricalcitol-treated mice. Although the frequencies of CD4^+^ T reg (CD4^+^Foxp3^+^) from paricalcitol were no higher than in the PDF group (*P* = .054), the absolute number of CD4^+^ Treg were significantly increased and CD4^+^ T cells from paricalcitol-treated mice appear to play a role in the prevention of PDF-induced fibrosis (Figure 3). In addition, CD4^+^ T cells and CD8^+^ T cells from the paricalcitol-treated mice, but not those from PDF mice, were able to reduce the frequency of IL-17^+^ T cells *in vitro* (Figure 7). This result clearly demonstrates that both, CD4^+^ and CD8^+^ T cells are able to regulate IL-17 production *in vitro*. The effect on IL-17 strongly correlated with decreased peritoneal fibrosis [37]. However, given that regulatory T cells and IL-17-secreting cells appear to have a common precursor, it does not exclude the possibility that, *in vivo*, VDR signaling could stimulate the differentiation into a regulatory T cell phenotype to the detriment of the Th17^+^ T cells. We cannot rule out the possibility that these mechanisms are working together.

VDR signaling has previously been related to fibroprotective activity [25], [26]. Various mechanisms have been proposed to explain the role of VDR signaling in fibrosis regulation, but it is still poorly understood. This study demonstrated that paricalcitol administered intraperitoneally reduced peritoneal membrane inflammation and fibrosis. The mechanism appears to be dependent primarily on the activation of Treg cells and the reduction of IL-17 secretion. These mechanisms are not mutually exclusive and might work simultaneously.

VDR is also expressed in macrophages, which could play a role in peritoneal fibrosis. Macrophages are the largest cell population in the peritoneal cavity. VDR signaling promotes macrophage differentiation into the M2 phenotype [43], which, as previously demonstrated, is related to peritoneal fibrosis in PD-treated patients [44]. In addition, the levels of TGF-β in the peritoneal cavity of the mice treated with paricalcitol were no different than those of the mice treated with PDF alone, and there was no correlation with peritoneal fibrosis. It has been suggested that the fibrotic activity of TGF-β is regulated by IL-17 and that inflammation-induced fibrosis could be prevented by targeting IL-17 [3434]. Therefore, at least in our model of PD-treated mice, the evidence suggests that VDR signaling in macrophages might not play a role in the prevention of peritoneal fibrosis.

Further studies will be required to elucidate other immunological mechanisms involved in peritoneal fibrosis. However, the results presented here strongly suggest that VDR signaling reduces peritoneal fibrosis through the augment of the number Treg and the downregulation of IL-17 production at peritoneum.

## Conclusion

VDR signaling promotes the recruitment of CD4^+^ and CD8^+^ T cells with regulatory activity into the peritoneal cavity, increasing their number, reducing IL-17 production and diminishing PDF-induced peritoneal inflammation and fibrosis. VDR signaling could be used to reduce peritoneal fibrosis induced by chronic exposure to PDF as well as surgery-induced adhesion.
